# Metastable States of Multiscale Brain Networks Are Keys to Crack the Timing Problem

**DOI:** 10.3389/fncom.2018.00075

**Published:** 2018-09-11

**Authors:** Tommaso Gili, Valentina Ciullo, Gianfranco Spalletta

**Affiliations:** ^1^IMT School for Advanced Studies Lucca, Lucca, Italy; ^2^Laboratory of Neuropsychiatry, IRCCS Santa Lucia Foundation, Rome, Italy; ^3^Department of Neurosciences, Psychology, Drug Research and Child Health, University of Florence, Florence, Italy; ^4^Division of Neuropsychiatry, Menninger Department of Psychiatry and Behavioral Sciences, Baylor College of Medicine, Houston, TX, United States

**Keywords:** brain networks, multiscale modeling, metastable state brain dynamics, timing and time perception, functional MRI, electrophysiology

## Abstract

The dynamics of the environment where we live in and the interaction with it, predicting events, provided strong evolutionary pressures for the brain functioning to process temporal information and generate timed responses. As a result, the human brain is able to process temporal information and generate temporal patterns. Despite the clear importance of temporal processing to cognition, learning, communication and sensory, motor and emotional processing, the basal mechanisms of how animals differentiate simple intervals or provide timed responses are still under debate. The lesson we learned from the last decade of research in neuroscience is that functional and structural brain connectivity matter. Specifically, it has been accepted that the organization of the brain in interacting segregated networks enables its function. In this paper we delineate the route to a promising approach for investigating timing mechanisms. We illustrate how novel insight into timing mechanisms can come by investigating brain functioning as a multi-layer dynamical network whose clustered dynamics is bound to report the presence of metastable states. We anticipate that metastable dynamics underlie the real-time coordination necessary for the brain's dynamic functioning associated to time perception. This new point of view will help further clarifying mechanisms of neuropsychiatric disorders.

## The view

Timing is an umbrella term that encompasses a variety of processes based on the prediction and estimation of temporal intervals across a wide range of scales, from hundreds of milliseconds to seconds. Theoretical models, mainly based on the existence of an internal clock (Gibbon, 1977), have been challenged by compelling behavioral findings that enhance suspects about its biological plausibility (Karmarkar and Buonomano, 2007). Alternate models have been proposed, describing timing as an ensemble of neural processes emerging from the activity of neural circuits inherently capable of temporal processing as a result of the complexity of cortical networks coupled with the presence of time-dependent neuronal properties (Buonomano and Maass, 2009). In this view, neural systems can benefit from the temporal evolution of their states, caused by the variation in neural and synaptic properties. The overall effect results in an adaptation of cerebral networks that could be tuned to discriminate temporal intervals (Bueno et al., 2017). State-dependent models can be extended to be consistent with the majority of timing models (Hass and Durstewitz, 2016), with the different models indicating specific constraints on what would collapse the state space. Although a route is traced toward a comprehensive description of timing, it is still unclear whether brain networks states are part of a coding scheme used to track time or a by-product of other processes that could generate a time-decodable signal. A possible theoretical framework could be the multi-scale description of brain networks both in space and time. On one hand it would be able to capture the local-to-global properties of neural processes that give rise to timing, on the other hand it would allow to grasp the integration processes among brain regions responsible for timing by means of metastability of network states (Friston, 1997; Fingelkurts and Fingelkurts, 2004, 2017; Deco and Kringelbach, 2016). Accordingly, our perspective view about the best strategy able to provide a coherent and complete description of timing can be divided in three steps: (1) the choice of tasks involving different aspects of timing (Coull and Nobre, 1998, 2008; Coull, 2004; Coull et al., 2013; Ciullo et al., 2018a) to be administered on a steady-state fashion (Gonzalez-Castillo and Bandettini, 2018; Tommasin et al., 2018) in order to saturate the activity of the areas interacting during the specific task; (2) the brain activity should be monitored by means of different techniques able to highlight different temporal and spatial scales (e.g., fMRI, hd-EEG, MEG). Specifically the different scales can be cast in a common framework according to the multilayer representation (De Domenico et al., 2013) (different spatial scales for the same time scale or different temporal scales for the same spatial one); (3) the temporal dynamics from each task will be finally analyzed and fitted to theoretical models of neuronal synchronization (Deco et al., 2017; Cavanna et al., 2018) in order to cluster the dynamics of brain's activity during time processing.

In the following paragraphs the core of each step is clarified and a review of the state-of-the-art is proposed.

## Timing in human and non—human animals

**Figure 1:**
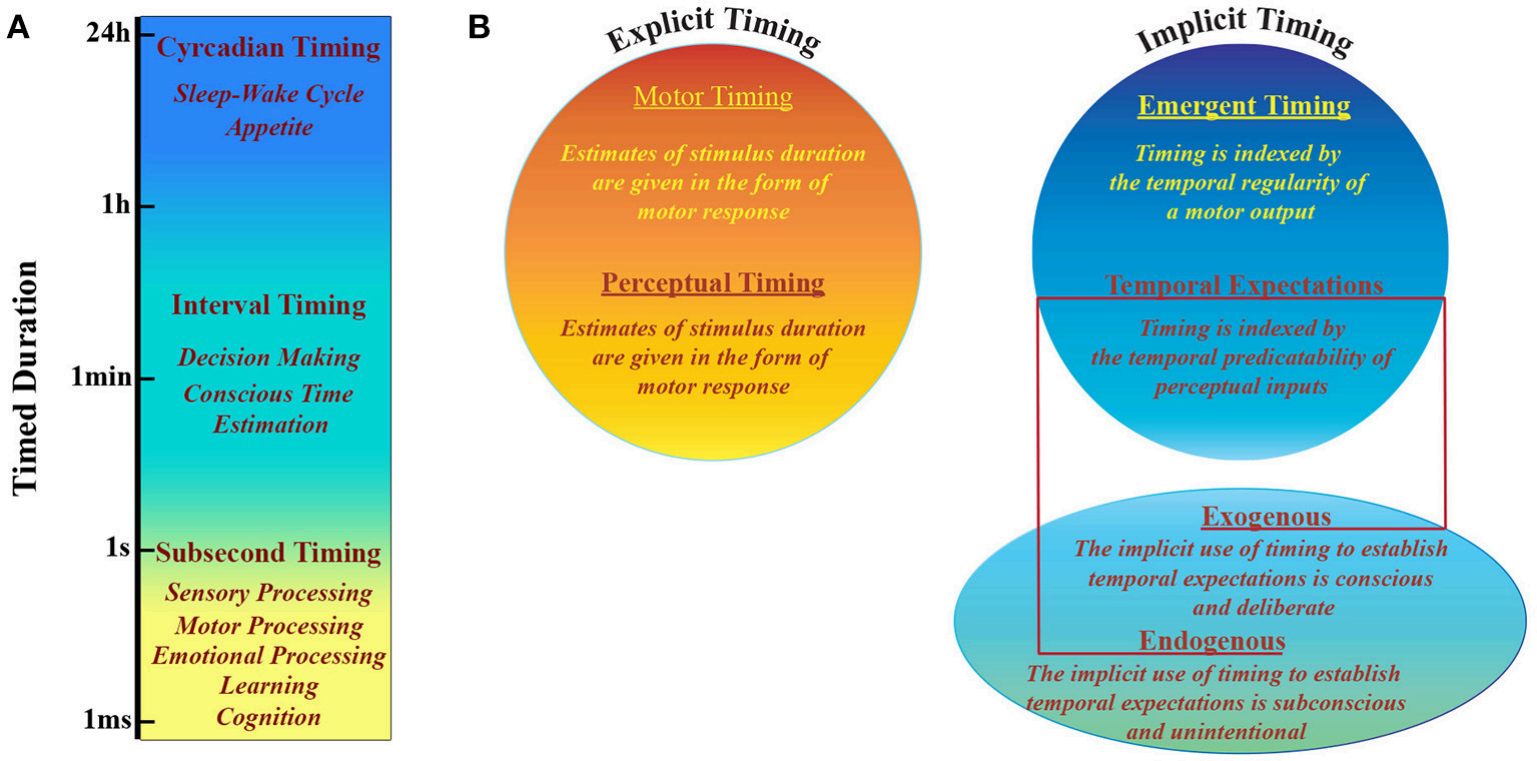
Timing taxonomy. **(A)** Human and non-human animals have developed multiple systems able to perform different tasks that are based on timing processing at different scales, that range over more than 10 orders of magnitude. **(B)** Explicit vs. Implicit timing. Explicit timing is engaged by tasks requiring either motor production (motor timing) or perceptual discrimination (perceptual timing) of a timed duration. Implicit timing is engaged as a product of the temporal regularity of either a motor output (emergent timing) or a perceptual input (temporal expectation). The latter can be established either incidentally via a temporally regular stimulus structure (exogenous temporal expectation) or deliberately via informative pre-cues (endogenous temporal expectation). Adapted from (Coull and Nobre, 2008).

The perception of what happens around us and the capacity to respond to it are crucially based on our ability of keeping track of time. Since both perception and action change over time, timing is necessary to estimate environmental dynamics, evaluate interplay between events and predict the consequences of our actions. Throughout normal development we acquire a sense of duration and rhythm that is basic to many behavioral aspects (Allman et al., 2012). Even if there is no specific system that senses time, human and non-human animals can estimate temporal intervals across a wide range of scales (Mauk and Buonomano, 2004; Buhusi and Meck, 2005). Intervals ranging from hundreds of milliseconds to seconds are typically associated with sensory and motor processing, learning, cognition and emotional processing (Figure 1), while larger intervals include processes that range from decision making to sleep-wake cycles (Buhusi and Meck, 2005). There is experimental evidence that timing is an intrinsic computational ability of every circuit in the cortex and that it can be performed locally. This notion implies that during perception tasks cortical networks can tell time as a result of time-dependent changes in synaptic properties, which influence any population response to sensory events in a history dependent fashion (Karmarkar and Buonomano, 2007). Furthermore, with the above mentioned sensory timing, motor timing is supposed to depend on the activity of highly connected cortical recurrent networks able to self-sustain activity (Mauk and Buonomano, 2004).

Psychophysical experiments suggest that sensory timing can be local (Johnston et al., 2006; Burr et al., 2007; van Wassenhove and Nagarajan, 2007), even if other results suggest that temporal performance variability in different contexts may be better described by a hybrid model (Merchant et al., 2008). Neuroimaging research suggests that a partially distributed timing mechanism sustains contextual flexibility. It is supposed to be integrated by core structures such as the cortico-thalamic-basal ganglia (CTBG) circuit and regions that are selectively engaged by different behavioral contexts (Buhusi and Meck, 2005; Coull et al., 2011). Cell activity changes, associated with temporal processing in behaving monkeys, have been described in areas composing different circuits responsible for sensorimotor processing via the skeletomotor or oculomotor effector systems (Perrett, 1998; Lebedev et al., 2007; Tanaka, 2007; Genovesio et al., 2009; Mita et al., 2009). Most of these studies reported climbing activity during different timing conditions: discrimination of time, time estimation, single interval reproduction and delay related response. Specifically, Merchant et al. (2013) showed a variable discharge rate of cells of Medial Premotor Cortex (MPC) as a function of interval durations with a synchronization-continuation tapping task. This suggested the MPC might contain a representation of interval duration, in the hundred of milliseconds, where diverse populations of interval-tuned cells are typically activated according to the duration of the produced interval. Ramping activity of MPC cells encodes either the elapsed or the remaining time for a temporalized movement such that the dynamic organization of motor intentions and action is sustained by ramping cells. Accordingly, interval tuning on the overall discharge rate affects more cognitive facets of temporal processing.

By moving to larger temporal and spatial scales, functional magnetic resonance imaging (fMRI) studies in humans showed that interval timing is regulated by distributed brain networks whose involvement is flexibly adapted according to task demands: timing emerges from the interaction among diverse brain regions rather than from processing in a specific one (Livesey et al., 2007; Coull et al., 2008; Harrington et al., 2010; Fingelkurts, 2014). For example pattern of timing-related activation in bilateral caudate and putamen was found to be distinguished from that found for most other brain regions in time-perception tasks. Only the anterior insula was found to exhibit the same activation pattern. This region crucially integrates processing from disparate domains (e.g., interoception, emotion, and cognition), including time (Kosillo and Smith, 2010; Wittmann et al., 2010), via its dense pattern of connections with most association areas in the basal ganglia and the occipital, temporal and prefrontal cortex. The connectedness of anterior insula with frontal cognitive control areas suggests that it supports the perceptual integration of sensory information (Eckert et al., 2009). By stimulating the supramarginal gyrus of the right hemisphere with transcranial magnetic stimulation (TMS) a dilation of perceived duration was induced because of its effect on interval encoding (Wiener et al., 2012). This result indicates that the neural circuitry that encodes time crucially includes the right supramarginal gyrus, confirming the detrimental effect of right parietal damage on time perception (Harrington et al., 1998). These findings support also the hypothesis of a network of multiple central clocks and distributed processes of timing mechanisms (Merchant et al., 2008). The ability to organize behaviors within periods in the range of seconds to minutes, depends on a cognitive system that requires multiple neuropsychological functions (Buhusi and Meck, 2005; Coull and Nobre, 2008), consequently pathophysiological distortions in time might reflect neuropsychological deficits typical of definite neuropsychiatric disorders as schizophrenia (Ciullo et al., 2016, 2018a), acquired brain injury (Piras et al., 2014), Parkinson's disease (Wearden et al., 2008), Huntington's disease (Beste et al., 2007) and attention-deficit hyperactivity disorder (Zelaznik et al., 2012). Thus, the understanding of timing mechanisms and of the related cognitive processes may also allow the realization of a model system aiming to characterize cognitive dysfunctions in order to define novel tools for early diagnosis and to develop novel targeted cognitive therapies. However, despite intensive investigations and substantial progress, the absence of a definitive framework encompassing the multifaceted nature of timing processes indicate that our understanding of the principles and mechanisms underlying brain functioning during time perception remains still incomplete. Nonetheless, all the results described above emphasize the role of interactions among distributed neuronal populations at different spatiotemporal scales in enabling flexible cognitive operations that give rise to sense of time (Fingelkurts and Fingelkurts, 2006). Given the functional specialization and integration that sustain the sense of time, a promising framework able to provide a modeling of time perception in the brain from an explicitly integrative perspective is represented by complex network theory. Recent developments in the quantitative analysis of complex networks, based largely on graph theory, have been rapidly translated to studies of brain network organization. Accordingly, the brain is described as a network of nodes and edges, while analytic advancements in network science and statistics allow us to represent and quantify functional interactions among brain regions of interest in order to make inferences about its organizational properties both at rest and as a function of cognitive demands. To our knowledge, a network based description of brain regions integration in timing is still largely incomplete and actually available only in Ciullo et al. (2018b) and Ghaderi et al. (2018).

This kind of cerebral systems modeling (Bassett and Sporns, 2017), will be crucially beneficial in the close future to an organic description of brain functioning during the estimation of temporal intervals and eventually to a better description of disorders characterized by impaired time perception.

## Multiscale brain networks

A tentative modeling of time perception processes necessarily points to a description of brain functioning based on the interplay of multi-scale brain networks (Fingelkurts et al., 2010; Bassett and Siebenhühner, 2013). The meaning of “scale” can vary according to the context: (i) a network's spatial scale, which refers to the resolution at which its connected regions of interest (nodes) and connections (edges) are defined, and can range from individual cells and synapses size (Jarrell et al., 2012; Shimono and Beggs, 2015; Lee et al., 2016), to brain regions and fiber tracts (Bullmore and Bassett, 2011) and (ii) temporal scales with precision ranging from sub-millisecond (Burns et al., 2014), to lifetime (Betzel et al., 2014; Gu et al., 2015). Although it is important to understand the functioning of individual elements, at each scale it is crucial to understand the sets of pair-wise relations that arrange the elements into the larger description of a totally interconnected system, namely the local and global topology of the network (Fingelkurts et al., 2010; Barabasi, 2016). Together these scales define a three-dimensional space in which the evolution of the brain network complexity is reported, being each point identified by three coordinates: space, time, and topology (Betzel and Bassett, 2017). Most descriptions of time perception mechanisms exist as single points in this space being based on analyses focused on networks defined singularly at one spatial, temporal, and topological scale. We anticipate that, while such studies have proven illuminating, in order to better understand the brain's true multi-scale, multi-level nature, it is essential that analyses begin to form bridges that link different scales to one another in order to offer a comprehensive description of the mechanisms that govern timing.

One promising approach to study a network that changes over multiple timescales is to make use of multi-layer network models of temporal networks (De Domenico et al., 2013; Kivelä et al., 2014). The multi-layer network model can treat estimates of the network's topology at different points of the time-scale as “layers.” This implies the necessity to integrate different modalities of investigation spanning different time-scales. It could be done by creating a multi-layer from different non-invasive neuroimaging techniques: from high-density electroencephalography (hd-EEG) (Liu et al., 2017), to magnetoencephalography (MEG) (de Pasquale et al., 2010), fast fMRI (Lewis et al., 2016), classical fMRI (Telesford et al., 2016) and combined EEG-fMRI (Mullinger and Bowtell, 2010; Yu et al., 2016). On the other hand, invasive approaches are able to detect multiple single neuron signals in non-human animals (Logothetis, 2012) and in human patients that need deep brain stimulation (Okun, 2014). Traditional analysis would characterize each layer independently of one another, while multi-layer network analysis treats the ensemble of layers as a single unit, characterizing its structure as a whole to explicitly bridge multiple temporal scales. Since the multi-layer network model doesn't depend on the timescales represented by each layer, it can include any timescale made accessible using neuroimaging technologies.

**Figure 2:**
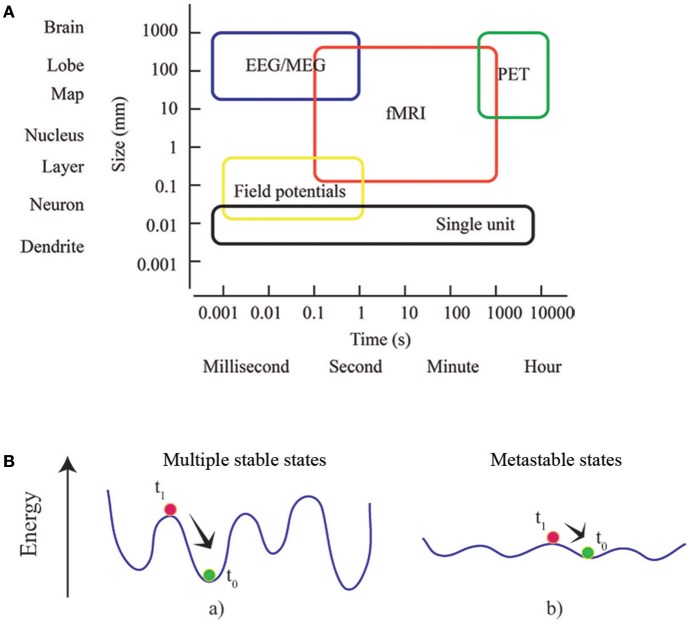
Multiscale and multistable nature of the brain. **(A)** Rather than considering the brain as a list of parts defined at a particular scale, brain network theory take advantage of the complexity of the interactions between the parts, and identifies the dependence of phenomena across scales. Box dimensions give outer bounds of the spatial and temporal scales at which relational data are measured and interactions unfold. Adapted from an image of neuroscience recording methods (Sejnowski et al., 2014). **(B)** The concepts of energy landscape and metastability. Points of these landscapes correspond to particular states of the system. The system at equilibrium (green) is perturbed (at t_0_) toward a state (red) that subsequently (at t_1_) relaxes. In (a) the system is stable and local minima (equilibrium points) are deep: dynamics are rapidly restored and the effects of perturbation are short-lasting. In (b) the energy landscape is almost flat and the stability of local minima decreases: the system can easily explore different (metastable) states without an external driving or endogenous fluctuation.

As well as for time, the space dimension can be also investigated at multiple scales (Figure 2A). MEG and fMRI analyses of human brain networks are limited by the accuracy of the inverse source localization of signal generators (MEG), and the spatial granularity of the individual voxel (fMRI). Nonetheless, it is possible to probe multiple spatial scales by appropriately aggregating the minimal units of interest into parcels or regions of interest. Several parcellation approaches have been proposed, distinguishing to one another according to different criteria as spatial variation in functional connectivity, myelination, cytoarchitectonics, etc. (Tzourio-Mazoyer et al., 2002; Craddock et al., 2012; Wang et al., 2015; Glasser et al., 2016; Gordon et al., 2016). Since the choice of parcellation condition*s* the network's topology (Wang et al., 2009; Zalesky et al., 2010), it must be checked if any result is not driven by the specific choice of parcellation, and is reproducible (at least qualitatively) using a different set of parcels at the same resolution (Bassett et al., 2011). A route for future research is to apply multi-scale topological analysis to voxel-level networks during the execution of tasks. It will allow identifying different parcels differentially involved in different brain states in order to sub-divide specific brain areas responsible for sustaining different cognitive engagements.

## Metastability: a resource of brain networks for sustaining time perception mechanisms

Large-scale brain networks have been showed to be organized according to multiple segregated sub-networks of interacting areas. It has been suggested that a dynamic, adaptable brain network arrangement in response to environmental stimulations underlies successful cognition (Bressler and Kelso, 2001; Fries, 2005). Dynamic combination of responses to sensory inputs, and spontaneous processing is at the core of brain activity, where task evoked responses should not be interpreted only in terms of localized processing, but should also take into account distributed processing occurring as activity flow across intrinsic networks (Smith et al., 2009; Zalesky et al., 2014; Sadaghiani et al., 2015; Cole et al., 2016; Shine et al., 2016). This allows a description of brain functioning in terms of a continuous recruitment of neuronal populations in a temporally coordinated fashion both during tasks execution, and at rest (Fingelkurts and Fingelkurts, 2005). Recently, it has been found that the neuronal engagement follows a precise hierarchy, according to two distinct sets of networks, or metastates, that the brain tends to cycle within (Vidaurre et al., 2017).

Metastates or metastable cerebral states are the core of a prominent conceptual framework known as Metastability (Scott Kelso, 1995; Fingelkurts and Fingelkurts, 2004, 2017; Freeman and Holmes, 2005; Werner, 2007). It offers a description of the reciprocal influence among interconnected parts and processes when pure synchronization does not occur. In coordination dynamics, such synchronization corresponds to stable fixed points of collective states (Friston, 1997). Metastability can be better understood by defining an energy landscape for the ensemble of possible states experienced by the brain: the phase space of the brain system (Fingelkurts and Fingelkurts, 2004, 2017). Generally, a system dynamically evolves attracted toward states of minimum energy, which can be either local or global. After being temporarily attracted toward a local state of minimum energy, an externally driven system can flee the basin of attraction and experience other equilibrium states. The dynamics of a metastable system is characterized by states that only transiently attract the dynamics. Since during its dynamic evolution the system tends to linger around these metastable states, the idea of a repertoire of conditions or configurations can be introduced (Figure 2B). Consequently, components are able to influence each other's destiny without being caught in a sustained state of synchronization, unable to create collectively new information Scott Kelso, 1995; Tognoli and Kelso, 2014). The emergence of metastable dynamics has been theoretically showed to be contingent upon the coupling between modules of a dynamical system (Friston, 1997; Strogatz, 2001; Shanahan, 2010; Cabral et al., 2011). Specifically, dynamic patterns of functional brain networks, consistent with metastable dynamics, come out when coupling is topologically characterized by short average path lengths and high clustering (Wildie and Shanahan, 2012) of modules. The efficiency of task-related brain activity has been showed to depend on metastability of spontaneous brain activity, which allows for optimal experience of the dynamical repertoire (Cabral et al., 2014). Recently metastability in brain networks has been investigated in aging, consciousness and neuronal communication in healthy subjects (Deco and Kringelbach, 2016; Deco et al., 2017; Naik et al., 2017; Cavanna et al., 2018) and in Schizophrenia and Alzheimer's disease patients (Córdova-Palomera et al., 2017; Koutsoukos and Angelopoulos, 2018). A variety of methods are described in order to capture synchronization and metastability in brain functioning.

Since metastability is a fundamental concept to grasp the behavior of complex systems theoretically and empirically, we anticipate that a form of metastability exists in time processing systems that parallels the metastability observed in many other aspects of brain functioning. The need for metastability in time perception modeling follows right from the definition. Metastability is the simultaneous occurrence of two competing tendencies: the inclination of individual components to exist as interacting entities and the propensity for the components to be characterized just by their independent behavior (Kelso, 2012). As a consequence metastability may be thought as a dynamical condition that allows the coordination of heterogeneous elements as it happens during time perception (brain areas having disparate intrinsic dynamics or brain areas whose activity is associated with different sensory, motor and cognitive processes) (Fingelkurts and Fingelkurts, 2006; Fingelkurts, 2014). Metastable brain theory may ameliorate timing modeling as it does not favor extremes, e.g., integrated vs. segregated processes, but it tends to reconcile them. Since metastability is a characteristic of the full complexity of the brain, it reaches a maximum when the balance between segregative and integrative forces is found. Furthermore, metastability doesn't need active induction since no disengagement mechanisms are required, as it happens in timing processing (Kononowicz et al., 2016). Finally, the crucial importance of time to perception and action necessitates metastability, in order to explain the ease with which timing can be performed by a range of different neural architectures. Clustering the dynamics of brain's activity during time processing may unearth the presence of metastable states associated with this specific aspect of cognition.

## Conclusion

Here we propose that the route along which future research will find novel insight into timing mechanisms is drawn in the direction of brain investigation as a multi-layer dynamical network whose clustered dynamics unavoidably reports the presence of metastable states. This perspective paves the way for future investigations into both the role of timing in other cognitive domains, from learning to agency, and the role that temporal dependency of brain network states has in cognition, elucidating the general characteristics of human cognitive activity that exists at a wide range of spatiotemporal scales. At the same time, our better understanding of dysfunctional timing processes will crucially allow us to develop novel diagnostics of neuropsychiatric diseases, and to design personalized therapeutics for rehabilitation and treatment of brain disorders characterized by distorted time perception.

## Author contributions

TG and GS conceived the paper. TG organized the paper. TG and VC wrote the first draft of the paper and collected and filtered the references. GS supervised the paper.

### Conflict of interest statement

The authors declare that the research was conducted in the absence of any commercial or financial relationships that could be construed as a potential conflict of interest.
